# Young people’s perspectives on vape access and policy: A qualitative study in Sheffield, England

**DOI:** 10.1016/j.puhip.2026.100871

**Published:** 2026-10-10

**Authors:** Calum Lewis, Hannah Fairbrother, Duncan Gillespie

**Affiliations:** School of Medicine and Population Health, University of Sheffield, Sheffield, United Kingdom

**Keywords:** e-cigarettes, Vapes, Vaping, Youth, Policy, Adolescence, Nicotine, Access, Public health, Tobacco control

## Abstract

**Objectives:**

This qualitative study aimed to explore young people’s experiences of accessing e-cigarettes (vapes) and to understand their perspectives on current and proposed UK vaping-related policy.

**Study design:**

Pragmatic qualitative study using two participatory workshops with five members of the Sheffield Youth Cabinet, aged 11 to 17, in Sheffield, England, March 2025. None of the participants reported vaping.

**Methods:**

Topic guides were informed by Penchansky and Thomas’s five dimensions of access (availability, accessibility, affordability, acceptability and accommodation). Workshops incorporated individual reflection and discussion. Data was audio-recorded, transcribed verbatim and analysed using Miles and Huberman’s thematic analysis, combining deductive coding using the access framework with inductive identification of emergent themes.

**Results:**

Five interconnected themes explained young people’s experiences of vape access and their ideas about access-related policy: (1) *A Flawed Information Environment*, where knowledge gaps and mistrust of official sources made vaping appear acceptable; (2) *The Social Normalisation of Vaping*, driven by peer behaviour, marketing and public visibility; (3) *The Appeal of Convenient Vaping*, with product design enabling discreet and frequent use; (4) *The Permissive and Affordable Retail Environment*, characterised by lax age-verification, proximity to schools and low cost; and (5) *Reframing Vapes as a Cessation Tool*, where participants suggested positioning vapes more clearly as cessation tools rather than consumer products. These themes reflected overlapping dimensions of access, showing that affordability, availability, accessibility, acceptability and accommodation interact rather than functioning independently.

**Conclusions:**

Participants described access to vapes as shaped by overlapping social, environmental and product-related factors that make vaping easy and appealing, which suggests that access is unlikely to be reduced by any single measure. They supported stronger controls on retail and marketing, but saw vapes as a way for adults to stop smoking. On that basis they opposed restricting flavours and proposed that vapes be positioned as cessation products.

**What this study adds**

• Provides qualitative evidence on how young people who do not vape describe the interacting regulatory, social and environmental factors shaping access to vapes.
• Shows how young people who do not vape interpret existing and proposed vaping policies, and how they understand their peers to adapt to and circumvent access restrictions.

**Implications for policy and practice**

• Single measures are unlikely to reduce access on their own, so policies should address several dimensions together.
• Restrictions on how vapes are marketed and sold could reduce their appeal to young people without closing off their use for smoking cessation, which is the balance participants proposed themselves.

## 1 Introduction

E-cigarettes (vapes) are used to support tobacco smoking cessation but have also become popular with non-smoking young people. [1], [2] This raises concerns about health risks, with an umbrella review finding associations between youth vaping and smoking initiation, substance use and respiratory outcomes. [3] Vaping uptake among young people who have never smoked is highest among young people living in contexts of socioeconomic deprivation, [4] the same groups in which smoking prevalence remains highest, [5] so any harms that do follow from vaping are likely to fall on the groups already most impacted by smoking. This creates a dilemma for policymakers: reducing vaping among young people who do not smoke without discouraging people who smoke from using vapes to cut down their smoking. [6]

Current vaping among 11- to 17-year-olds in Great Britain rose from 3.2% in 2021 to a peak of 7.6% in 2023 and has since fallen slightly to 6.4% in 2026, with 4% vaping more than once a week. [7] In response to this the United Kingdom (UK) government banned disposable vapes in June 2025, [8] and introduced the Vaping Products Duty in October 2026. [9] The Tobacco and Vapes Act also received Royal Assent in April 2026, [10] which gives the government powers over the packaging and retail display of vaping products, over branding and imagery that appeals to children, and over the flavour descriptors that may be used, though how these powers will be used is still being decided.

Drivers of young people’s vaping include marketing, [11], [12] peer norms, [13], [14] low cost, [13], [15], [16] retailer proximity, [13], [15], [16] weak age-of-sale enforcement [12], [17], discreet product design [18], [19] and inaccurate harm perceptions. [7] Evidence on how policy affects those drivers remains limited, particularly real-world evidence on regulations targeting products, prices and retail practices. [20] Participatory work with young people can help by showing how vapes are obtained and how policy changes may be experienced in practice. [21] Our recent systematic review conceptualised access using five dimensions (availability, affordability, accommodation, accessibility and acceptability [22]) that shape young people’s ability to obtain and use vapes. [23] This study aimed to refine that understanding using young people’s perspectives. We had two objectives:

(1) To explore young people’s experiences of vape access to identify the key factors that policy could address.
(2) To capture young people’s perspectives on proposed UK policy options and potential unintended consequences.

## 2 Methods

### 2.1 Qualitative approach

This study followed a pragmatic qualitative design with participatory workshops. [24] The lead researcher facilitated two workshops with the same group. Workshop topic guides were informed by the access framework from our recent review [23] (see Table 1).

**Table 1:** Dimensions of access.

Dimension of access	Penchansky & Thomas (1981) Definition [22]	Study Definition
Affordability	The relationship between the price of services and the client’s ability to pay, including indirect costs such as transportation and lost wages.	The price of vapes in relation to a young person’s ability to pay. This includes the direct cost of products, such as cheap disposable vapes, and the relative cost compared to alternatives like tobacco cigarettes. Affordability is influenced by policies such as taxation, which directly target product price, and restrictions on availability of certain products that may also be low-cost options.

Acceptability	The relationship between clients’ attitudes about personal and practice characteristics of providers and providers’ attitudes about acceptable personal characteristics of clients. This includes issues such as age, sex, ethnicity, and social status.	How well-aligned vapes are with young people’s personal preferences and social norms, and how this alignment influences their decision to use them. This could include social desirability as influenced by perceptions of peer use, and perceptions of the health harms of vapes. Industry primarily influences acceptability through advertising, including point-of-sale displays and online promotions.

Accommodation	The manner in which the supply of healthcare services is organised to accept clients, including appointment systems, hours of operation, and telephone services.	How easily young people can use vapes in various settings. This is influenced by policies creating vape-free spaces (e.g., in schools, on public transport) and the degree to which people are aware of these rules and adhere to them. It is also affected by inherent product characteristics, such as device size and minimal odour, which enable discreet use in prohibited areas.

Availability	The volume and type of existing services and resources in relation to clients’ needs. This includes supply factors such as sufficient personnel, facilities, and equipment.	The overall supply of vapes accessible to young people through both formal and informal channels. This is influenced not only by market-level legislation like product bans (e.g., on disposables) or increases in the Minimum Legal Sales Age, but also by informal social networks, such as sourcing from older siblings, friends, or peers.

Accessibility	The relationship between the location of supply and the location of clients, considering travel time, distance, and transportation options.	The ease with which young people can obtain vapes from commercial retail sources. This includes the density and proximity of physical retailers (e.g., vape shops, convenience stores), which can be influenced by policy measures like retailer licensing schemes, and the ease of purchasing through online channels. It is significantly influenced by retailer practices, such as the enforcement (or lack thereof) of age-of-sale verification at the point of purchase.

### 2.2 Ethics

Ethical approval was obtained from the University of Sheffield’s School of Medicine and Population Health Ethics Committee (Reference Number: 058481).

### 2.3 Sampling and recruitment

Participants were members of the Sheffield Youth Cabinet (SYC), a youth advisory group of 11- to 17-year-olds elected by their peers from across Sheffield, United Kingdom. The Cabinet draws its membership from a range of ethnic and socioeconomic backgrounds, and its members are accustomed to discussing policy-relevant issues affecting young people. Five members of the SYC took part in this study. All five attended the first workshop and three returned for the second. None reported vaping. Beyond age range and sex, no demographic information was collected. Participants received a £50 voucher across the two sessions, an amount set in line with NIHR INVOLVE guidance [25] and approved as part of the ethical review.

### 2.4 Data collection methods

Two 2-h workshops were co-designed with SYC staff, audio-recorded and transcribed by the lead researcher (CL). The workshops took place in March 2025, while the Tobacco and Vapes Bill was before Parliament and three months before the ban on disposable vapes came into force. The first opened with a presentation on the concept of access and explored perceptions of vaping; the second opened with a presentation on UK Government proposals and focused on policy options. [26] In both sessions, participants were given the opportunity to reflect individually in notebooks before taking part in a facilitated group discussion. However, very little was written in the notebooks, and the analysis therefore draws only on the transcripts. At the start of the second workshop CL presented a summary of the initial analysis from the first session and asked participants whether it matched what they remembered and whether anything was missing. The group confirmed the summary cohered with their recollection and raised no additions, so the initial themes were carried into the second phase of coding unchanged.

### 2.5 Data analysis

Thematic analysis followed the process of data reduction, data display, and conclusion drawing and verification developed by Miles and Huberman. [27] Data reduction involved two phases of coding in NVivo. An initial deductive codebook used the dimensions of access (see Table 1) as main codes, with known themes and policy options as sub-codes. A second round of inductive coding identified recurring themes not captured in the codebook, including the role of information and the reframing of vapes as a medical product. Codes were then organised into a diagram to visualise interrelationships, producing five overarching themes, and transcripts were revisited to find supporting evidence for them. Coding and theme development were conducted by one researcher (CL), with emerging themes and uncertainty discussed with the research team.

## 3 Results

Five themes explained how young people experience access to vapes and their ideas about policy options (see Table 2).

**Table 2:** Key themes, access dimensions and related policy measures.

Theme	Dimensions of access	What participants described	Policy measures discussed
A Flawed Information Environment	Acceptability	Gaps in knowledge about contents and addiction, combined with distrust of official sources, made vaping appear less risky and more acceptable	Health information and campaigns

The Social Normalisation of Vaping	Acceptability	Marketing, peer and family use, and visible vaping in public places made the behaviour appear usual	Advertising restrictions; packaging, branding and flavour descriptor controls

The Appeal of Convenient Vaping	Accommodation	Vapes need no preparation, can be used indoors and are easily concealed, so use fits settings where smoking would not, which also makes it hard to police	Vape-free places

The Permissive and Affordable Retail Environment	Availability, Accessibility, Affordability	Vapes were seen as cheap and easily obtained from local shops with weak age checks, and through friends and siblings	Vaping Products Duty; retailer licensing; age-of-sale enforcement

Reframing Vapes as a Cessation Tool	Availability, Acceptability	Participants proposed positioning vapes as cessation aids rather than consumer products, with supply linked to healthcare settings	Restrictions on retail availability; advertising restrictions

### 3.1 A flawed information environment

#### 3.1.1 Knowledge gaps and perceptions of health risks

Participants suggested that knowledge gaps around health risks can make vaping seem desirable. They suggested that young people often begin vaping without understanding addiction risks, ‘ … no one goes into vaping like, “oh, I’m going to get addicted”’ [Workshop 1, Female]. Participants reported limited awareness amongst young people about the contents of vapes:

*‘If you ask most people actually, do you even know what you’re inhaling? Most of them would just be like, “yeah, cherry”’* [Workshop 1, Male].

Conversely, participants noted that greater awareness of health impacts could prompt quitting, describing peers who stopped once they understood the effects. They suggested clearer information on the contents and risks of vapes is needed, with one stating, ‘ … there isn’t enough advertisement on what’s actually in there … ’ [Workshop 1, Male].

#### 3.1.2 Dissemination of information and trust

School assemblies were described as ineffective because students do not pay attention and social acceptance outweighs official warnings:

*‘we do not care about what the teacher is talking about in assembly … as long as people see them [vapes] as cool they are going along with it. They don’t care [about] assemblies and stuff … ’* [Workshop 1, Female].

The effectiveness of health messaging was hampered by mistrust of official sources, with some participants believing government motives were financial: ‘I don’t think they actually are doing anything to stop it because they just want money’ [Workshop 1, Female]. Their doubt was about the source of the information, not its accuracy, and they expected no meaningful action to follow. Their alternative suggestion was to fund independent research into the harms of vapes from tax revenue on vape sales.

### 3.2 The social normalisation of vaping

#### 3.2.1 Marketing, advertising, and product design

Participants reported that vapes are deliberately marketed to young people rather than to adults who smoke, pointing to bright colour schemes and what they called ‘childish names’ for flavours, like ‘apple bursting’ [Workshop 1, Female]. They also highlighted the retail environment’s role in normalisation, describing vapes displayed in shops alongside ‘biscuits then like chocolate and like bubble gum’ [Workshop 1, Female]. The same participant supported moving vapes out of open display, suggesting they ‘should be like on the other side of the counter all the time’ [Workshop 1, Female].

Social media was discussed as a platform for unregulated marketing. Participants noted that algorithms could push vaping content to young users, while content like vape tricks and influencer promotion made vaping appealing outside of formal advertising. Participants supported restrictions on marketing, suggesting ‘banning things like the names of vapes because … names are probably bigger than watching a commercial’ [Workshop 2, Female]. However, they acknowledged the complexities of social media regulation, such as controlling unlabelled influencer content and the ease with which young users bypass age restrictions. Despite these calls for restrictions, participants did not support a ban on flavours, viewing them as important for helping people who smoke quit, stating it would be ‘taking away the only good thing vapes have’ [Workshop 2, Female].

#### 3.2.2 Social and environmental exposure

In addition to marketing, participants suggested normalisation is driven by exposure within peer and family groups: *‘It’s simply how much exposure you have to certain things … How much are they around you? How much do you perceive it as a normal thing?’* [Workshop 1, Male].

Friends’ vaping was cited as a trigger for others’ vaping initiation, framed as a ‘cool and trendy group activity’ [Workshop 1, Male]. Vaping was also perceived to spread from popular people at school. Similarly, participants suggested having parents who vape could normalise the behaviour from a young age. However, they also described a reverse normalisation effect, where increased prevalence led some peer groups to view vaping as ‘silly’ [Workshop 1, Female].

#### 3.2.3 Visibility and policy considerations

Participants reported that vaping in public spaces normalises the behaviour for young people. When vape-free zones were discussed, young people noted that designated vaping areas may not solve the issue if they are in visible public spaces. Instead, participants suggested less visible vaping areas and banning vaping in cars with children, with one reasoning, ‘if you don’t want your children to do bad things then don’t do it in front of them’ [Workshop 2, Female].

### 3.3 The appeal of convenient vaping

#### 3.3.1 Product design

Participants felt the design of vapes makes them preferable to cigarettes, contrasting the effort of rolling a cigarette and having to go outside with a vape that can be used continuously and indoors:

*‘When somebody has a cigarette … it takes ages to roll and you can only do it outside, [so] you can only have 5 or 10 a day. However, if you have a vape, you can’t stop yourself. It’s just like taking a drink of water, in-out’* [Workshop 1, Female].

Furthermore, participants reported that vapes were easier to conceal from parents as, ‘you can just chuck them straight in the bin’ [Workshop 2, Female].

#### 3.3.2 The challenges of enforcement in schools

What made vapes convenient also made it difficult to police their use in schools. Barriers to control included the number of students vaping, with one participant saying there was ‘no point in trying to stop them’ [Workshop 1, Female], the use of hard-to-monitor areas like toilets, and inconsistent enforcement by teachers, who were described as ranging from ‘super chill’ to ‘absolutely strict’ [Workshop 1, Male].

### 3.4 The permissive and affordable retail environment

#### 3.4.1 Retailer practices and proximity

Participants reported that despite age-of-sale laws, vapes were easily accessible from local convenience or corner shops, which were perceived as having lax age verification: ‘most shops just don’t care. They’ll sell them to young children’ [Workshop 1, Female]. One participant remarked that it was ‘easier to buy vapes than candy sometimes’ and described shops near their school as holding ‘an abundant amount of vapes’ compared with shops elsewhere [Workshop 1, Male]. Supermarkets and specialist vape stores were seen as enforcing stricter checks. Participants supported retailer licensing, which they felt would deter corner shops from illicit sales, a ban on selling vapes near schools, and fines for employees who sell to underage customers.

#### 3.4.2 Low cost

Participants identified affordability as a driver of young people’s vaping, compounding the issue of retailer practices and proximity. Price increases were seen as an effective deterrent, with one noting that if a vape cost the ‘equivalent of three lunches’ [Workshop 1, Female], young people would reconsider. Cost also influenced device choice, with cheaper disposables described as a low-commitment entry point for people new to vaping or vaping socially.

#### 3.4.3 Social networks and proxy purchasing

Alongside shops, participants described informal supply through older friends and siblings. This could evolve from a favour into a regular practice, with one participant describing an older sister of a school peer who ‘was making money off these kids and she didn’t even know them’ [Workshop 1, Female]. Other routes included parents buying for their children, online sales via Snapchat, and sharing a single device among a group.

### 3.5 Reframing vapes as a cessation tool

When discussing policy solutions, participants suggested regulating vapes like medical drugs could change how they are perceived and used, making young people ‘realise the seriousness of it’ [Workshop 2, Female]. Health practitioners could prescribe vapes to help people ‘wean yourself off’ [Workshop 2, Female] cigarettes, paired with a ban on general advertising, with any remaining marketing restricted to healthcare settings: ‘Maybe they should keep advertisement however … it has to be in place of like rehab’ [Workshop 2, Female]. They suggested this could make vapes seem less like ordinary consumer products and more like cessation tools.

## 4 Discussion

This study identified several interconnected factors that young people described as facilitating vape use, including social normalisation, product appeal, low cost and easy retail access. These findings align with our recent review, which found that young people’s access to vapes is shaped by interacting social, commercial and regulatory factors. [23]

Participants described vapes as cheap and easily obtained from local shops where age checks were perceived as lax, consistent with UK research showing corner shops are seen as easier to buy from than supermarkets or vape shops. [28] Retail environments shape consumption of unhealthy commodities more broadly, [29] and young people recognise these influences and favour structural responses. [30] The Tobacco and Vapes Act now confers enforcement and retailer licensing powers which could be targeted at the shops participants identified as least likely to check age, [10] and the Vaping Products Duty will raise prices, although its effect may be weakened if industry pricing keeps some products affordable, as has been observed for tobacco. [31], [32] The disposable vape ban may have indirectly addressed cost by targeting the cheapest category of vapes, [8] but manufacturers have since introduced rechargeable products resembling disposables in appearance and price. [33]

Participants highlighted how product design, branding, retail displays and the sight of others vaping contributed to the appeal and apparent normality of vaping. Bright colours and flavour names reinforced the perception that the products were aimed at younger audiences, consistent with evidence that flavour descriptions increase appeal among adolescents, [34] and devices that were easy to use and conceal fitted readily into daily life. UK research shows young people are more likely than adults to notice vape displays in shop windows and to view them positively. [28] The packaging and display powers under the Tobacco and Vapes Act, [10] which are currently being consulted on, [35] may reduce youth appeal while having less impact on people trying to quit cigarettes.

Participants’ sense of how common vaping was among their peers was higher than in recent prevalence data. One described vaping in their school as being so widespread, particularly in older year groups, that there was no point in trying to stop it, but national figures suggest that even among the oldest pupils, only about 13% currently vaped in 2026. [7] Adolescents elsewhere have similarly described vaping as more common among peers than surveys record, and it is this perceived prevalence that shapes how normal a behaviour appears. [36] However, participants also described peer groups in which rising visibility had made vaping seem less desirable, so these beliefs are not fixed. This study cannot show how far perceptions influence behaviour, but approaches that provide a clear picture of the prevalence of vaping may be worth considering alongside measures aimed at the behaviour itself.

Participants expressed uncertainty about both the addictive nature of vapes and their health risks, describing peers who began vaping without expecting to become addicted and who knew little about what they were inhaling. They wanted greater transparency about ingredients and product contents as a deterrent but doubted the motives of the government and schools that would provide it. This suggests current health messaging may not be addressing the specific questions young people have. A key challenge, however, is that messaging aimed at young people also reaches adults who smoke. Among these adults, 54% now believe vaping is at least as harmful as smoking, a decline in accurate understanding that Action on Smoking and Health (ASH) attribute partly to coverage of youth vaping. [37] Adults who hold this belief are more likely to relapse to smoking after quitting, [6] so stronger warnings aimed at young people could unintentionally work against adult cessation. Information about product contents and addiction is closer to what participants asked for and may be less likely to distort how vaping compares with smoking.

A novel finding was the participant-led suggestion that vapes should be positioned as smoking cessation tools rather than consumer products, with retail availability restricted and access linked to healthcare settings. The appeal of this to participants was that it would make vaping seem serious rather than recreational. Adults who vape have described medicalised approaches as offering reassurance about the safety of vaping relative to smoking, while raising concern that restricting supply to medical routes would narrow the product choice associated with successful switching. [38] UK policy already promotes vapes as cessation aids, such as through the Swap to Stop programme, [39] although the dominant model remains widespread retail availability. However, more restrictive models can carry their own risks, with Australia’s pharmacy-only policy associated with the growth of illicit markets. [40]

Participants also identified ways in which the measures they discussed might have unintended consequences. They opposed restricting flavours, which they saw as the feature making vapes useful to adults trying to stop smoking. National survey data suggests a further difficulty, since a substantial minority of young people who vape said they would seek illegal flavours or stockpile ahead of such a restriction. [7] On vape-free zones, young people in our study suggested designated areas in visible public places might sustain the visibility those measures are designed to reduce. They also noted that promotion reaches young people through algorithmic recommendations on social media rather than formal advertising, which a ban on advertising may not be able to cover. In each case participants saw a reason the measure might not achieve what it intends, whether because it would work against efforts to support adult cessation or because it misses the way young people encounter vapes.

This study has several limitations. The small sample size facilitated in-depth discussion, but limits generalisability to all young people across the UK. As SYC members, participants may have been more policy-engaged than their peers. None reported vaping, so while they described what they saw around them, they could not speak to the experience of using vapes themselves. The workshops predate the disposable vapes ban, the Vaping Products Duty and the Tobacco and Vapes Act, so participants were describing a market that has since changed, particularly in the availability of the cheap disposables they identified as an entry point. Furthermore, these findings only apply to the UK tobacco and nicotine context and these markets differ internationally. For example, products like snus [41] and nicotine pouches [42] vary in popularity and regulation, as well as vaping policy itself differing between countries. [43], [44]

In conclusion, young people in our study described cost, retail practice, product appeal and social visibility as overlapping rather than separate, so that access is unlikely to be reduced by any single measure. These participants recognised the same trade-off policy is trying to resolve, between restricting a consumer market and preserving a route out of smoking. They opposed flavour restrictions on that basis and proposed that vapes be positioned as cessation products rather than consumer goods.

## Ethical approval and informed consent statements

Ethical approval was obtained from the University of Sheffield’s School of Medicine and Population Health Ethics Committee (Reference Number: 058481). For participation and publication written consent was obtained from participants aged 16 years and older. Legal guardian consent was obtained for participants aged under 16 years.

## Data availability statement

Underlying research materials are available from the corresponding author on reasonable request.

## Funding statement

This research was funded in whole, or in part, by the Wellcome Trust [228353/Z/23/Z]. For the purpose of Open Access, the author has applied a CC BY public copyright licence to any Author Accepted Manuscript version arising from this submission.

## Declaration of competing interest

The authors declare that they have no known competing financial interests or personal relationships that could have appeared to influence the work reported in this paper.
